# The OfJAZ3-OfMYB21 complex mediates jasmonic acid signaling pathway to regulate linalool biosynthesis in *Osmanthus fragrans*

**DOI:** 10.1093/hr/uhaf321

**Published:** 2025-11-25

**Authors:** Yangang Lan, Xue Huang, Ziyi Li, Shunran Zhang, Yan Xiang, Hongbo Zhao

**Affiliations:** School of Landscape Architecture, Zhejiang Agriculture and Forestry University, Hangzhou 311300, China; Anhui Provincial Key Laboratory of Forest Resources and Silviculture, Anhui Agricultural University, Hefei 230036, China; School of Landscape Architecture, Zhejiang Agriculture and Forestry University, Hangzhou 311300, China; School of Landscape Architecture, Zhejiang Agriculture and Forestry University, Hangzhou 311300, China; Anhui Provincial Key Laboratory of Forest Resources and Silviculture, Anhui Agricultural University, Hefei 230036, China; Anhui Provincial Key Laboratory of Forest Resources and Silviculture, Anhui Agricultural University, Hefei 230036, China; School of Landscape Architecture, Zhejiang Agriculture and Forestry University, Hangzhou 311300, China

## Abstract

*Osmanthus fragrans* is a well-known ornamental tree species for its pleasing floral fragrance. Linalool, as the characteristic aromatic component of *O. fragrans*, holds significant potential for applications in the flavor and fragrance industry. Although jasmonic acid (JA) is well documented to regulate the biosynthesis and accumulation of various plant secondary metabolites, its role in linalool biosynthesis remains largely unclear. Here, we discovered a positive correlation between the endogenous JA levels and linalool accumulation during the flowering stage of *O. fragrans*. Exogenous JA treatment was shown to enhance linalool biosynthesis by activating the linalool synthase gene *OfTPS2*. Dual-LUC and EMSA assays demonstrated that the key protein in the JA signaling pathway, OfJAZ3, interacted with OfMYB21 and subsequently suppressed the transcriptional activation of *OfTPS2* mediated by OfMYB21. Functional validation further revealed that overexpression of *OfJAZ3* significantly inhibited linalool biosynthesis in *O. fragrans*, *A. thaliana*, and *N. tabacum* plants. In contrast, JA promoted the degradation of OfJAZ3, thereby disrupting the formation of the OfJAZ3-OfMYB21 complex and relieving its inhibitory effect on *OfTPS2*. Split-LUC, BiFC, and pull-down assays confirmed that OfJAZ3 interacted with the F-box protein OfCOI1 (a key component of the E3 ubiquitin ligase SCF^COI1^ complex), and JA treatment enhanced the strength of this interaction. Moreover, OfCOI1 was found to participate in *OfTPS2* regulation by facilitating the ubiquitination and degradation of OfJAZ3. In conclusion, our findings elucidate the molecular mechanism by which OfJAZ3-OfMYB21 complex mediates JA signaling to regulate linalool biosynthesis in *O. fragrans*.

## Introduction

*Osmanthus fragrans* is extensively cultivated as an ornamental landscape species, valued for its prolific flowering and distinctive floral fragrance [1, 2]. Beyond its visual ornamental value, the floral volatile compounds of *O. fragrans* have significant commercial applications in the food, cosmetics, and phytotherapy industries [3, 4]*.* The monoterpenoid linalool has been identified as the dominant characteristic volatile organic compound in *O. fragrans* flowers, consistently present across different cultivars [5–8]. Moreover, as a high-value industrial and food additive, global linalool consumption exceeds 1000 metric tons annually [9]. Understanding the biosynthetic pathway and regulatory network of linalool could therefore offer innovative biotechnological approaches to improve floral fragrance characteristics in plants while boosting production efficiency of economically important terpenoid compounds.

Recent advances in multi-omics approaches have enabled the systematic investigation of linalool biosynthesis pathways in *O. fragrans*, facilitating the identification of key terpene synthase genes and their transcriptional regulators [8, 10, 11]. Zeng *et al.* identified four terpene synthase (TPS) genes in *O. fragrans*, among which *OfTPS1* and *OfTPS2* were functionally characterized as linalool synthases [12]. In a complementary study, Xiong *et al.* discovered two additional TPS genes in *O. fragrans* using integrated transcriptomic profiling and in vitro enzymatic assays; among them, one was molecularly validated as a linalool synthase and was similarly designated *OfTPS2* [13]. Comparative studies demonstrated that aroma differences between the *O. fragrans* cultivars are driven by the differential expression of two genes: *CCD4* and *LIS1*. Specifically, CCD4 mediates β-ionone production via carotenoid degradation, while LIS1 regulates the synthesis of linalool precursors [14]. Regarding transcriptional regulation, Ding *et al.* carried out genome-wide identification and expression profiling of WRKY transcription factors in *O. fragrans* [15]. Notably, the expression patterns of *OfWRKY7/19/36/139* showed significant correlations with the emission levels of distinct monoterpenes. In a concurrent study, Li *et al.* identified 243 MYB transcription factors in *O. fragrans* [16]; of these, *OfMYB19/20* expression levels were positively correlated with linalool oxide accumulation, while *OfMYB51/65/8/121/137/144* exhibited negative correlations with multiple linalool oxides. Consistent with these findings, we previously found that the R2R3-MYB transcription factor OfMYB21 activates its expression by binding to the *OfTPS2* promoter, actively regulating the biosynthesis of linalool, and preliminarily revealing the molecular mechanism of *O. fragrans* enrichment of linalool [17].

The jasmonic acid (JA) signaling pathway plays a pivotal role in modulating the biosynthesis of plant secondary metabolites [18]. In *Gossypium arboreum*, JA enhances the expression of *GaWRKY1* and the sesquiterpene synthase gene *CAD1-A*, thereby promoting sesquiterpenoid biosynthesis [19]. Similarly, *A. thaliana* JAZ proteins and DELLA proteins physically interact with the transcription factor MYC2, forming a regulatory complex that suppresses MYC2-mediated transcriptional activation [20]. Upon exogenous JA or GA application, JAZ and DELLA proteins undergo degradation, releasing MYC2 to activate TPS genes and subsequently increasing sesquiterpenoid production. The bioactive JA-Ile is perceived by the SCF^COI1^ ubiquitin ligase complex, comprising the F-box protein COI1 and JAZ repressors. This recognition triggers the ubiquitination and 26S proteasome-mediated degradation of JAZ proteins, leading to the release of downstream transcription factors and activation of JA-responsive genes [21]. In *Salvia miltiorrhiza*, JA regulates the biosynthesis of medicinal metabolites via the JAZ9-MYB76 complex [22]. Our preliminary studies have demonstrated that exogenous JA treatment enhances linalool biosynthesis in *O. fragrans* by up-regulating *OfTPS2* expression [17]. Meanwhile, the key protein OfJAZ3 in the JA signaling pathway interacts with the upstream regulatory factor OfMYB21 of *OfTPS2*. However, the molecular mechanism of OfJAZ3 and the involved JA signaling pathway in the biosynthesis of linalool *O. fragrans* have not been elucidated.

In this study, we found that during the flowering process of *O. fragrans*, the JA levels are closely associated with the biosynthesis of linalool. Moreover, JA treatment activates the expression of *OfTPS2* to promote linalool biosynthesis. The key repressor protein OfJAZ3 in the JA signaling pathway forms a transcriptional complex by interacting with OfMYB21, thereby inhibiting the OfMYB21-mediated activation of *OfTPS2* and the subsequent biosynthesis of linalool. JA treatment facilitates the ubiquitination and degradation of OfJAZ3, which disrupts the OfJAZ3-OfMYB21 protein complex and releases OfMYB21 to activate *OfTPS2*. *In vivo* and *in vitro* protein–protein interaction assays revealed OfCOI1, an F-box protein of the E3 ubiquitin ligase complex in the JA signaling pathway, interacts with OfJAZ3. OfCOI1 participates in the regulation of *OfTPS2* by facilitating ubiquitination-dependent degradation of OfJAZ3. Collectively, our results elucidate the molecular mechanism by which OfJAZ3 mediates JA signaling to regulate linalool biosynthesis in *O. fragrans*.

## Results

### JA positively regulates linalool biosynthesis in *O. fragrans* by activating *OfTPS2*

**Figure 1:**
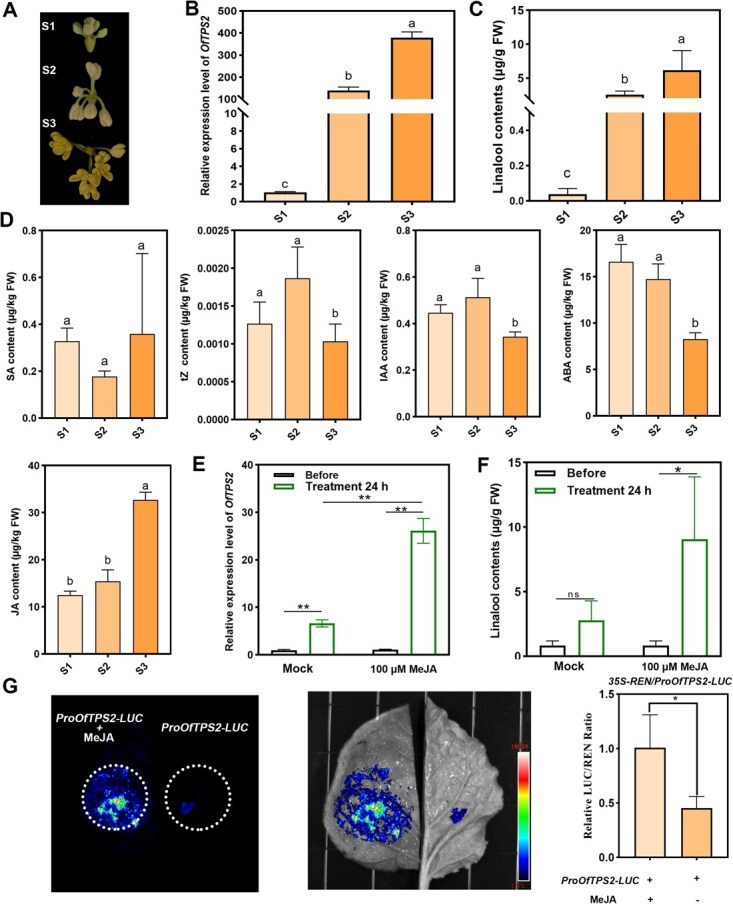
JA plays a crucial regulatory role in linalool biosynthesis in *O. fragrans*. (A) Three flowering stages of *O. fragrans*: S1 (bud stage), S2 (initial flowering stage), and S3 (full flowering stage). (B) Expression levels of the linalool synthase gene *OfTPS2* across the three flowering stages of *O. fragrans*. (C) Linalool biosynthesis levels in *O. fragrans* at the three flowering stages. (D) Quantitative determination of five endogenous hormones (SA, tZ, IAA, ABA, and JA) at three key flowering stages of *O. fragrans*. (E) Determination of *OfTPS2* expression levels in *O. fragrans* flowers under exogenous MeJA treatment; flowers treated with the solvent used to dissolve MeJA served as the control. (F) Determination of linalool biosynthesis levels in *O. fragrans* flowers under exogenous MeJA treatment. (G) Relative LUC to REN ratios from transient expression assays. These represent the activity of the *OfTPS2* promoter in the absence/presence of MeJA. Error bars indicate the SD of three biological replicates, and the asterisk represents the significant difference, evaluated by the Student’s *t*-test (^*^  *P* < 0.05, ^**^  *P* < 0.01). Different letters above the bars indicate significant differences between groups (*P* < 0.05).

To investigate the regulatory role of endogenous hormones in linalool biosynthesis in *O. fragrans* flowers, this study selected three key flowering stages (designated as S1, S2, and S3) to detect the expression of key genes and the content of volatile compounds (Fig. 1A–C). Meanwhile, liquid chromatography-mass spectrometry (LC–MS) was used for quantitative determination of five endogenous hormones, including salicylic acid (SA), trans-zeatin (tZ), indole-3-acetic acid (IAA), abscisic acid (ABA), and JA (Fig. 1D). The results showed that during the transition from S1 to S3, both the expression level of the linalool synthase gene *OfTPS2* and linalool biosynthesis were significantly up-regulated. Among the five endogenous hormones, only the content of JA increased significantly during this transition, which was consistent with the trend of linalool biosynthesis levels. To further validate the causal link between JA and linalool biosynthesis in *O. fragrans*, S1-stage flowers were exogenously treated with 100 μM methyl jasmonate (MeJA), a bioactive derivative of JA. MeJA treatment remarkably induced *OfTPS2* transcription, and subsequent metabolic profiling demonstrated that linalool production was substantially enhanced compared to the mock-treated control group (Fig. 1E and F). Furthermore, Dual-LUC reporter assays coupled with *Nicotiana benthamiana* transient expression systems provided direct evidence for the activating effect of MeJA on *OfTPS2* expression (Fig. 1G). Collectively, these findings establish that JA modulates linalool biosynthesis in *O. fragrans* through a regulatory pathway targeting the linalool synthase gene *OfTPS2*, highlighting the critical role of JA signaling in mediating terpenoid metabolism during floral development.

### OfJAZ3, a JA signaling repressor, inhibits *OfTPS2* transcriptional activation mediated by OfMYB21

The role of JA in linalool biosynthesis in *O. fragrans* prompted us to further explore the underlying molecular regulatory mechanisms. In our previous study, we identified that the transcription factor OfMYB21 positively regulates linalool biosynthesis in *O. fragrans* by activating *OfTPS2* [17]. Moreover, overexpression of *OfMYB21* significantly enhanced linalool biosynthesis in *A. thaliana* and *N. tabacum*, which confirms the conserved function and core role of OfMYB21 in linalool biosynthesis (Supplementary Fig. S1). Concurrently, our preliminary work also demonstrated that the JAZ protein OfJAZ3 interacts with OfMYB21; however, the detailed regulatory mechanism of this interaction remains elusive. Given that OfJAZ3 serves as a key repressor in the JA signaling pathway, which motivated us to further investigate the regulatory role of OfJAZ3 in linalool biosynthesis in *O. fragrans*.

**Figure 2:**
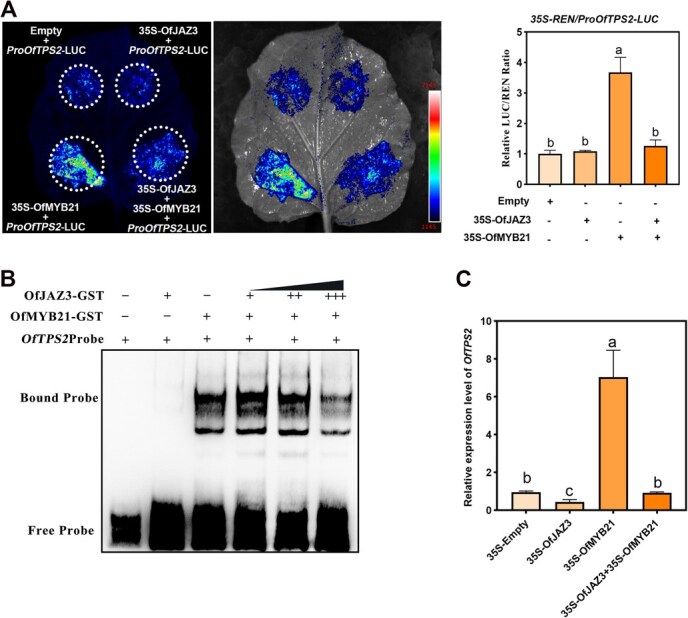
OfJAZ3 inhibits the transcriptional activation of the linalool synthase gene *OfTPS2* by OfMYB21. (A) Dual-luciferase reporter gene assay validation shows that co-transformation of OfJAZ3/OfMYB21 significantly suppresses the promoter activity of *OfTPS2* compared to transformation with OfMYB21 alone. (B) EMSA validation demonstrates that OfJAZ3 inhibits the binding of OfMYB21 to the MYB-binding site on the *OfTPS2* promoter. (C) The transient expression system in *O. fragrans* flowers was used to validate the regulatory effect of the OfJAZ3-OfMYB21 transcriptional complex on *OfTPS2* expression. Different letters above the bars indicate significant differences between groups (*P* < 0.05, Student’s *t*-test).

To further clarify the regulatory role of the OfJAZ3-OfMYB21 transcriptional complex, we employed a Dual-LUC assay to investigate the effect of OfJAZ3 on the promoter activity of *OfTPS2*. The results showed that when OfMYB21 was co-transformed with the reporter gene driven by the *OfTPS2* promoter into *N. benthamiana* plants, OfMYB21 exhibited an activating effect on the *OfTPS2* promoter; in contrast, the sole expression of OfJAZ3 failed to activate the *OfTPS2* promoter. However, when OfJAZ3 and OfMYB21 were co-transformed, the transcriptional activation of *OfTPS2* by OfMYB21 was significantly attenuated (Fig. 2A). The EMSA results demonstrated that when GST-OfJAZ3 fusion protein was incubated with the biotin-labeled *OfTPS2* promoter sequence probe and subjected to gel electrophoresis, no retarded bands were observed (Fig. 2B). On the contrary, a shifted band was detected when the GST-OfMYB21 recombinant protein was used instead. Furthermore, when the GST-OfMYB21 and GST-OfJAZ3 recombinant proteins were co-incubated with the biotin-labeled probe, the intensity of the shifted band was reduced, and this reduction became more pronounced with the increase in the concentration of the GST-OfJAZ3 recombinant protein, indicating a weakened binding affinity of OfMYB21 to the *OfTPS2* promoter. Subsequently, using a transient expression system in *O. fragrans* flowers, we further found that when *OfJAZ3* and *OfMYB21* were co-overexpressed in *O. fragrans* flowers (Supplementary Fig. S2), the expression level of *OfTPS2* was significantly lower than that when *OfMYB21* was overexpressed alone (Fig. 2C). Collectively, these findings demonstrate that OfJAZ3 interacts with OfMYB21, thereby repressing the transcriptional regulation of *OfTPS2* by OfMYB21.

### OfJAZ3 inhibits the biosynthesis of linalool in *O. fragrans*, *A. thaliana*, and *N. tabacum* plants

**Figure 3:**
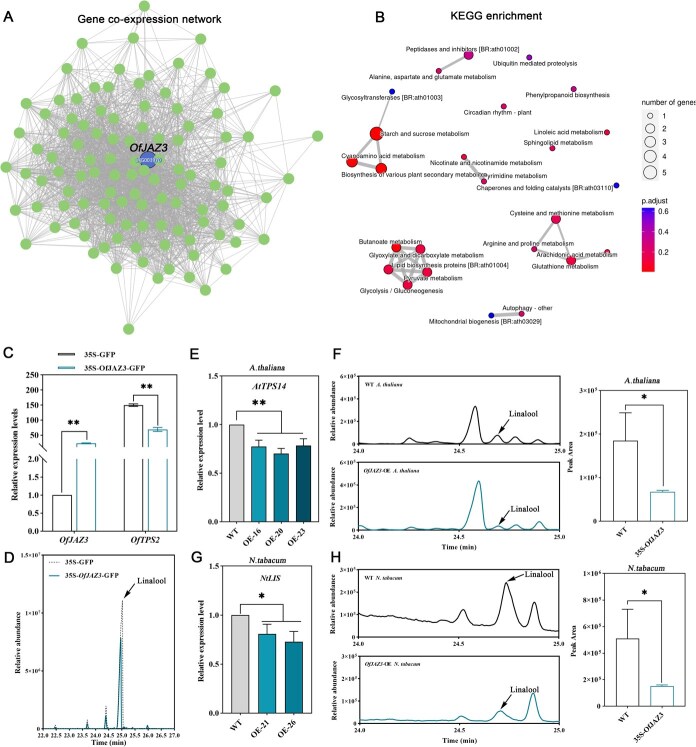
OfJAZ3 inhibits the biosynthesis of linalool. (A) Gene co-expression network involving *OfJAZ3*. (B) KEGG pathway enrichment analysis for genes co-expressed with *OfJAZ3*. (C) Transient overexpression of *OfJAZ3* in *O. fragrans* flowers significantly up-regulates *OfTPS2* expression. (D) Comparison of linalool abundance between flowers expressing 35S::GFP and 35S::OfJAZ3-GFP in *O. fragrans*. (E) Detection of the expression levels of the linalool synthase gene *AtTPS14* in WT and *OfJAZ3*-overexpressing transgenic *A. thaliana* plants. (F) Comparison of linalool abundance between WT and *OfJAZ3*-overexpressing transgenic *A. thaliana* plants. (G) Detection of the expression levels of the linalool synthase gene *NtLIS* in WT and *OfJAZ3*-overexpressing transgenic *N. tabacum* plants. (H) Comparison of linalool abundance between WT and *OfJAZ3*-overexpressing transgenic *N. tabacum* plants. Error bars indicate the SD of three biological replicates, and the asterisk represents the significant difference, evaluated by the Student’s *t*-test (^*^  *P* < 0.05, ^**^  *P* < 0.01).

Given that the JA signaling repressor OfJAZ3 is involved in the regulation of *OfTPS2*, we further analyzed the function of OfJAZ3 in linalool biosynthesis. We constructed a gene co-expression network containing *OfJAZ3* (Fig. 3A) and performed KEGG pathway enrichment analysis on genes co-expressed with *OfJAZ3*. KEGG enrichment results revealed that genes co-expressed with *OfJAZ3* were significantly enriched in pathways such as ‘Biosynthesis of various plant secondary metabolites’ and ‘Ubiquitin mediated proteolysis’ (Fig. 3B), suggesting that OfJAZ3 plays a role in terpenoid biosynthesis and its protein may be modified by ubiquitination. To verify the regulatory function of OfJAZ3 in linalool biosynthesis, we transiently overexpressed *OfJAZ3* in *O. fragrans* flowers using the transient transformation technology. The results indicated that transient overexpression of *OfJAZ3* in *O. fragrans* flowers significantly down-regulated the expression of *OfTPS2* (Fig. 3C). Furthermore, GC–MS analysis revealed that compared with the control group, transient overexpression of *OfJAZ3* in *O. fragrans* flowers reduced the abundance of linalool (Fig. 3D). In the *A. thaliana*, the overexpression of *OfJAZ3* markedly repressed the expression of the *AtTPS14* (Fig. 3E), subsequently resulting in a significant decrease in the linalool abundance among the volatiles emitted by the flowers of this transgenic line (Fig. 3F). Analogously, in *OfJAZ3*-overexpressing transgenic *N. tabacum* plants, the expression level of the *NtLIS*, a key enzyme for linalool synthesis, was also notably suppressed (Fig. 3G). This alteration directly manifested as a pronounced reduction in linalool accumulation in the leaves of the transgenic *N. tabacum* plants (Fig. 3H), robustly demonstrating that the overexpression of *OfJAZ3* effectively diminished the linalool synthetic capacity in these transgenic plants.

### JA restored the transcriptional activity of OfMYB21 by inducing degradation of OfJAZ3

**Figure 4:**
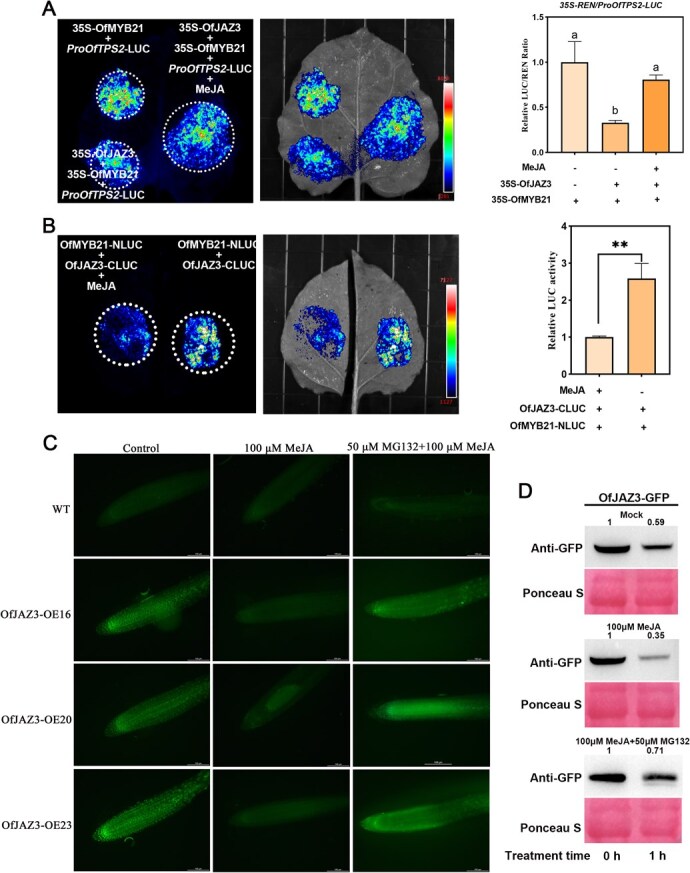
JA restored the transcriptional activity of OfMYB21 by inducing degradation of OfJAZ3. (A) Dual-luciferase reporter gene assay analysis of the regulatory effect of OfJAZ3-OfMYB21 on the *OfTPS2* promoter under exogenous MeJA treatment. (B) Split luciferase (Split LUC) assay combined with exogenous MeJA treatment to examine the modulation of JA on the interaction intensity between OfJAZ3 and OfMYB21. (C) Detection of GFP fluorescence signals in root tips of 35S::OfJAZ3-GFP transgenic *A. thaliana* plants under exogenous MeJA treatment. (D) Cell-free degradation assay to assess the impact of JA on the stability of the OfJAZ3 protein. Error bars indicate the SD of three biological replicates, and the asterisk represents the significant difference, evaluated by the Student’s *t*-test (^*^  *P* < 0.05, ^**^  *P* < 0.01). Different letters above the bars indicate significant differences between groups (*P* < 0.05).

Here, the Dual-LUC assays revealed that when OfJAZ3 and OfMYB21 were co-transformed into *N. benthamiana* leaves, OfJAZ3 significantly suppressed the activating effect of OfMYB21 on the *OfTPS2* (Fig. 4A). Notably, under exogenous MeJA treatment, the inhibitory effect of OfJAZ3 was abrogated (Fig. 4A). Split LUC assays demonstrated that *N. benthamiana* leaves co-transformed with OfJAZ3-cLuc and OfMYB21-nLuc exhibited robust LUC fluorescence signals in the co-transformation region (Fig. 4B and Supplementary Fig. S3). However, exogenous treatment with MeJA solution led to a significant reduction in LUC activity, implying that MeJA attenuates the interaction between OfJAZ3 and OfMYB21. As key regulators in the JA signaling pathway, the stability of JAZ proteins is modulated by JA signals. To examine whether the stability of the OfJAZ3 protein is regulated by JA, we examined the GFP fluorescence in the roots of 7-day-old transgenic seedlings (35S::OfJAZ3-GFP transgenic *A. thaliana* plants) under both MeJA-treated (2 h) and untreated conditions (Fig. 4C). In the absence of MeJA, a nuclear-localized GFP signal was observed in all 35S::OfJAZ3-GFP transgenic plants. However, treating the seedlings with 100 μM MeJA resulted in the loss of nuclear 35S::OfJAZ3-GFP signal, although a diffuse fluorescence pattern was still detected in the roots. Furthermore, pretreatment of seedlings with MG132, a specific 26S proteasome inhibitor, hindered the MeJA-induced degradation of OfJAZ3-GFP, suggesting that the 26S proteasome pathway mediates the JA-induced degradation of OfJAZ3. To investigate the hormone-dependent stability of OfJAZ3, we employed a cell-free assay system to assess whether the protein levels of OfJAZ3 are regulated by JA, through transient expression analysis of *N. benthamiana* leaves expressing GFP-tagged OfJAZ3. The results demonstrated that treatment with MeJA facilitated the degradation of OfJAZ3-GFP protein (Fig. 4D).

### OfJAZ3 interacts with the F-box protein OfCOI1, a key component of the E3 ubiquitin ligase SCF^COI1^ complex

**Figure 5:**
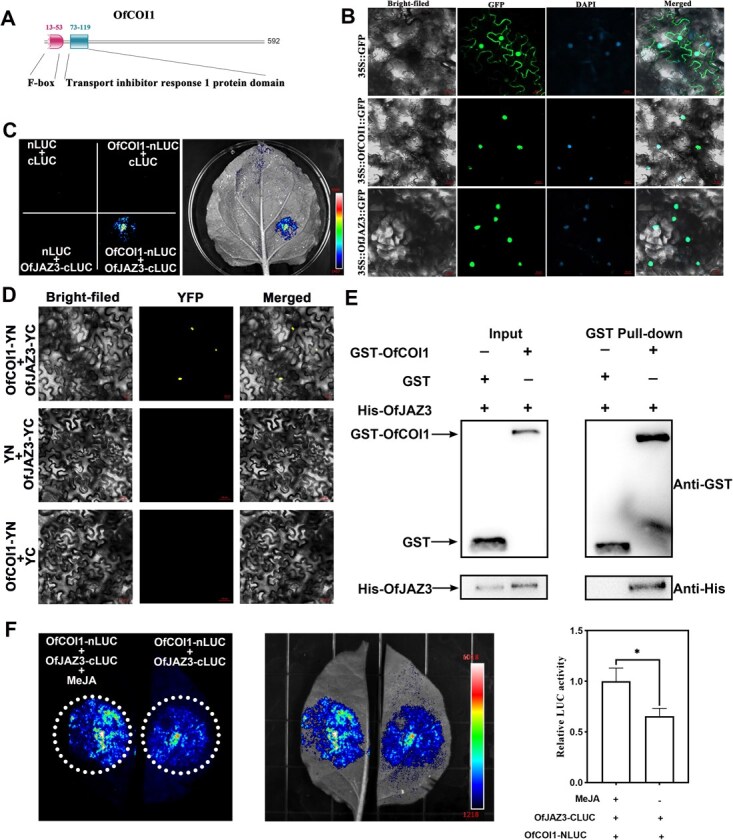
The E3 ubiquitin ligase OfCOI1 interacts with OfJAZ3. (A) Protein domain analysis of OfCOI1. (B) Subcellular localization of the OfCOI1 and OfJAZ3 protein. (C) Split luciferase assay confirmation of the interaction between OfCOI1 and OfJAZ3 in *N. benthamiana* leaf cells. (D) Bimolecular fluorescence complementation (BiFC) assay validation of the interaction between OfCOI1 and OfJAZ3 in *N. benthamiana* leaf cells. (E) GST pull-down assay verification of the interaction between OfCOI1 and OfJAZ3. (F) Split LUC assays confirmed that MeJA treatment enhances the interaction strength between OfCOI1 and OfJAZ3. Error bars indicate the SD of three biological replicates, and the asterisk represents the significant difference, evaluated by the Student’s *t*-test (^*^  *P* < 0.05, ^**^  *P* < 0.01).

Previous studies have shown that the F-box protein CORONATINE INSENSITIVE1 (COI1) is a component of the E3 ubiquitin ligase complex Skip-Cullin-F-box (SCF^COI1^). JA and its active form JA-Ile can trigger the formation of a complex between COI1 and JAZ, thereby inducing the degradation of JAZ in a COI1-dependent manner via the 26S proteasome. Here, we identified an F-box protein in *O. fragrans* that exhibits high homology to *A. thaliana* COI1 using multiple sequence alignment, and named it OfCOI1 (Fig. 5A). Subcellular localization experiments revealed that OfCOI1 is localized to the nucleus, consistent with the localization of OfJAZ3 (Fig. 5B). Split-LUC assays demonstrated that OfCOI1 interacts with OfJAZ3 (Fig. 5C). To further validate the interaction between OfCOI1 and OfJAZ3, we performed BiFC assays (Fig. 5D). The results showed that co-transformation of OfCOI1-nYFP and OfJAZ3-cYFP resulted in yellow fluorescent protein (YFP) signals in the nuclei of *N. benthamiana* leaf cells. Next, we performed in vitro pull-down assays using a GST-OfCOI1 recombinant protein and a His-OfJAZ3 fusion protein. When incubated with GST-OfCOI1, the anti-GST antibody pulled down His-OfJAZ3, but not when incubated with GST alone (Fig. 5E). Split LUC assays revealed that co-expression of OfCOI1-nLUC and OfJAZ3-cLUC in *N. benthamiana* leaves produced strong luciferase activity (Fig. 5F). Interestingly, exogenous MeJA treatment further enhanced the interaction strength between OfCOI1 and OfJAZ3, which was manifested by a significant increase in luminescent signals (Fig. 5F). Taken together, these results confirm that there is a physical interaction between OfCOI1 and OfJAZ3, and that MeJA treatment enhances the strength of their interaction.

### OfCOI1 mediates the ubiquitination and degradation of OfJAZ3, thereby restoring the transcriptional activation of *OfTPS2* by OfMYB21

**Figure 6:**
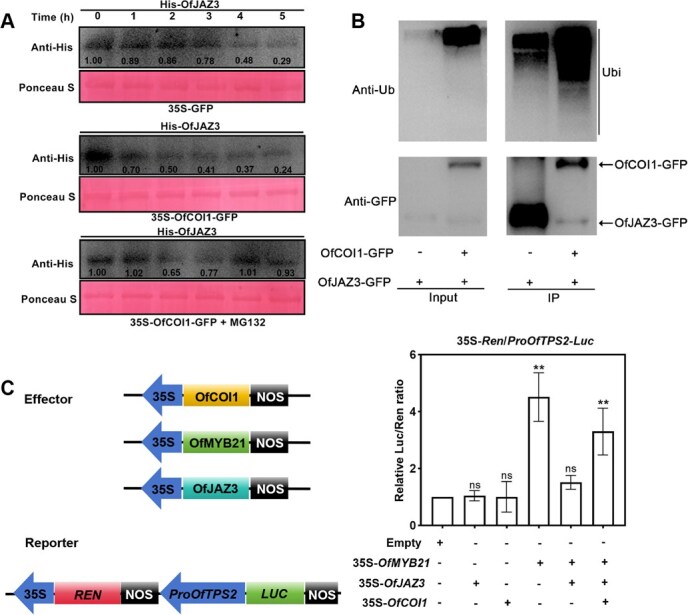
OfCOI1 mediates the ubiquitination of OfJAZ3 to restore the transcriptional activation of *OfTPS2* by OfMYB21. (A) OfCOI1 promotes proteasome-mediated degradation of OfJAZ3. Using an anti-His antibody, the levels of His-OfJAZ3 in total crude extracts from 35S::GFP and 35S::OfCOI1-GFP transgenic *N. benthamiana* leaves, incubated at 4°C for the indicated time periods and treated with or without 50 μM MG132, were analyzed. (B) In vivo ubiquitination analysis of OfJAZ3. The recombinant vector 35S::OfJAZ3-GFP was transiently expressed alone or co-expressed with 35S::OfCOI1-GFP in *N. benthamiana* leaves. The leaves were treated with MG132 for 12 h prior to sampling. Immunoprecipitation (IP) products obtained using an anti-GFP antibody, as well as crude extracts (Input), were analyzed by immunoblotting with anti-GFP and anti-ubiquitin antibodies, respectively. (C) The dual-luciferase reporter gene assay demonstrates that OfCOI1 restores the transcriptional activation of *OfTPS2* by OfMYB21. Error bars indicate the SD of three biological replicates, and the asterisk represents the significant difference, evaluated by the Student’s *t*-test (^*^  *P* < 0.05, ^**^  *P* < 0.01).

To further investigate whether OfCOI1 regulates the stability of the OfJAZ3 protein, we conducted in vitro protein degradation assays (Fig. 6A). Immunoblot analysis revealed that the OfJAZ3 protein degraded more rapidly in total protein extracts from 35S::OfCOI1-GFP *N. benthamiana* leaves compared to those from 35S::GFP *N. benthamiana* leaves. Moreover, MG132 reduced the degradation of OfJAZ3 in the total protein extracts from 35S::OfCOI1-GFP *N. benthamiana* leaves. These results indicate that OfCOI1 interacts with OfJAZ3 and promotes its degradation via a proteasome-mediated degradation pathway. To investigate whether OfCOI1 promotes the ubiquitination of OfJAZ3, we transformed the constructed 35S::OfJAZ3-GFP or 35S::OfJAZ3-GFP/35S::OfCOI1-GFP vectors into *N. benthamiana* leaves to express OfJAZ3-GFP and OfJAZ3-GFP/OfCOI1-GFP proteins, respectively. After extracting total proteins, immunoprecipitation was performed using anti-GFP and anti-ubiquitin antibodies. As shown in the figure, the immunoprecipitated samples were analyzed with anti-ubiquitin and anti-GFP antibodies. The results revealed that the level of polyubiquitination was significantly enhanced in the OfJAZ3-GFP/OfCOI1-GFP *N. benthamiana* leaf samples, indicating that OfCOI1 promote the ubiquitination of OfJAZ3 (Fig. 6B). Subsequently, we further employed the dual-LUC assays to investigate whether OfCOI1 could influence the regulatory effect of OfJAZ3 on *OfTPS2*. The results demonstrated that OfJAZ3 inhibited the transcriptional activation of *OfTPS2* by OfMYB21, whereas OfCOI1 rescued the activation of *OfTPS2* by OfMYB21 through the degradation of OfJAZ3 (Fig. 6C).

## Discussion

Terpenoids are a class of natural products synthesized by plants with significant biological functions and economic value [23]. As a representative monoterpene, linalool serves as a core component of floral volatile organic compounds (VOCs) in aromatic plants, and exhibits enormous application potential in industries such as food, pharmaceuticals, and household chemicals [24–26]. Therefore, elucidating the biosynthetic and regulatory mechanisms of linalool is crucial for improving floral aroma quality and advancing research on terpenoid metabolic engineering. Studies have demonstrated that R2R3-MYB transcription factors regulate terpenoid metabolism by directly binding to the promoters of terpene synthase (TPS) genes [27, 28]. Previous studies from our group identified *OfTPS2* as the linalool synthase gene in *O. fragrans* and revealed that its upstream regulator OfMYB21 activates *OfTPS2* expression by binding to MYB motifs within its promoter. In this study, heterologous overexpression of *OfMYB21* in *A. thaliana* and *N. tabacum* further confirmed that OfMYB21 enhances linalool biosynthesis by up-regulating endogenous *TPS* homologs. This regulatory mechanism is conserved across different species. For instance, FhMYB21L2 in *Freesia hybrida* activates monoterpene synthesis by binding to MYBCORE motifs in the *FhTPS1* promoter [9], while AmMYB24 in *Antirrhinum majus* regulates the expression of ocimene synthase gene *AmOCS* via MYBCOREATCYCB1 motifs [29].

Phytohormones have emerged as key regulators of linalool biosynthesis [30]. However, it remains unclear whether the biosynthesis of linalool in *O. fragrans* is regulated by its endogenous hormones. Our study identified JA as a critical signaling molecule modulating linalool production in *O. fragrans*. During the early stage of *O. fragrans* flower development, the endogenous JA content exhibited a consistent trend with the expression level of *OfTPS2* and the linalool accumulation. Furthermore, exogenous JA treatment was found to regulate linalool biosynthesis by activating the expression of *OfTPS2*, though the underlying molecular mechanism requires further investigation. As key proteins in the JA signaling pathway, JAZ proteins in *A. thaliana* inhibit floral organ development by interacting with AtMYB21/AtMYB24 [20]. In contrast, overexpression of the JAZ transcription factor OsJAZ8 in rice suppresses linalool synthesis and increases the plant’s susceptibility to bacterial leaf streak [31]. Using Dual-LUC assays and EMSA experiments, we demonstrated for the first time that OfJAZ3 in *O. fragrans* specifically inhibits the transcriptional activation of *OfTPS2* by forming a repressive complex with OfMYB21. This negative regulatory mechanism is homologous to the JAZ-MYB21/24 module involved in *A. thaliana* floral development, suggesting a conserved regulatory network for terpenoid metabolism across plants. Furthermore, transient overexpression of *OfJAZ3* in *O. fragrans* flowers, as well as stable overexpression in transgenic *A. thaliana* and *N. tabacum* plants, significantly down-regulated TPS gene expression and reduced linalool accumulation. These findings are consistent with the functional role of OsJAZ8 in rice, further confirming the cross-species conservation of JAZ-mediated terpenoid regulation. However, how JA regulates linalool biosynthesis through modulating OfJAZ3 remains unknown.

The mechanism by which JA signaling regulates JAZ protein stability via the ubiquitin-proteasome system (UPS) has been characterized in multiple species [32]. For example, in *Salvia miltiorrhiza*, JA induces 26S proteasome-dependent degradation of SmJAZ9, thereby relieving its inhibition on tanshinone biosynthesis [22]. In apple, JAZ proteins suppress anthocyanin synthesis by disrupting the MBW complex, while exogenous JA treatment promotes JAZ degradation and activates pigment accumulation [33]. Our study revealed that exogenous JA accelerates OfJAZ3 degradation and weakens its interaction with OfMYB21, whereas the proteasome inhibitor MG132 blocks this process. These results clarify the molecular pathway by which JA signaling regulates OfJAZ3 stability via UPS. This discovery mechanistically links with JA-induced terpenoid synthesis in *Camellia sinensis* [34] and *Sindora glabra* [35], providing novel insights into the universal regulatory principles of JA in terpenoid metabolism.

**Figure 7:**
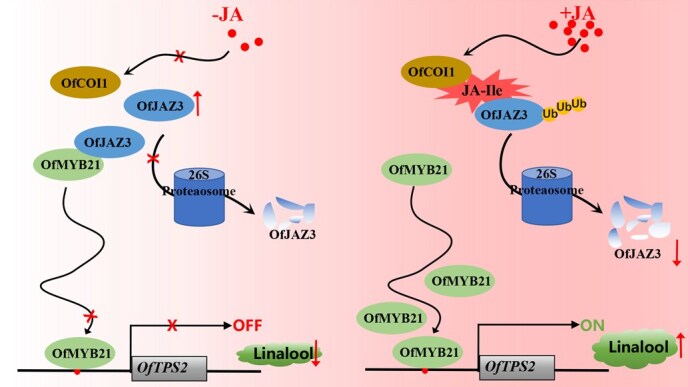
A hypothetical model diagram illustrating the involvement of the OfJAZ3-OfMYB21 complex in the regulation of linalool biosynthesis through the jasmonic acid signaling pathway.

Here, we identified OfCOI1 in *O. fragrans*, a homolog of *A. thaliana* COI1, and confirmed its in vivo interaction with OfJAZ3. This finding directly connects the canonical JA signaling pathway with terpenoid metabolism, laying the foundation for elucidating the molecular mechanism of SCF^OfCOI1^-mediated OfJAZ3 degradation. Notably, E3 ligase-mediated targeting of transcription factors holds significant value for metabolic engineering. For example, the apple E3 ligase MdMIEL1 inhibits anthocyanin synthesis by ubiquitinating MdMYB308L for degradation [36], while the banana E3 ligase MaXB3 delays fruit ripening by degrading MaNAC029 [37]. These examples highlight the potential of future research to investigate OfCOI1-mediated ubiquitination of OfJAZ3 and its regulatory effects on linalool synthesis, which may offer new targets for metabolic engineering in aromatic plants. Based on the above results, we propose a hypothetical model diagram (Fig. 7) for JA-mediated linalool biosynthesis: JA treatment promotes the interaction between OfCOI1, a key protein of the E3 ubiquitin ligase, and OfJAZ3, which subsequently induces the degradation of OfJAZ3 via the ubiquitination pathway to release OfMYB21. Consequently, OfMYB21 activates *OfTPS2* to facilitate linalool biosynthesis.

## Conclusion

In summary, based on the results of this study, we propose a regulatory model illustrating how the OfJAZ3-OfMYB21 module mediates the JA signaling pathway to regulate linalool biosynthesis in *O. fragrans* (Fig. 7). Specifically, when OfJAZ3 interacts with the transcription factor OfMYB21 to form a protein complex, it attenuates the transcriptional activation of the *OfTPS2* gene by OfMYB21, thereby inhibiting linalool biosynthesis. In contrast, increased endogenous or exogenous JA levels induce the interaction between the E3 ubiquitin ligase SCF^COI1^ complex and OfJAZ3, which promotes the ubiquitination and degradation of OfJAZ3 via the 26S proteasome pathway. This disruption of the OfJAZ3–OfMYB21 interaction ultimately restores the transcriptional activation of *OfTPS2* by OfMYB21.

## Materials and methods

### Plant materials and treatments

*Osmanthus fragrans* (*O. fragrans* cultivar ‘Liuyejingui’), Arabidopsis (*A. thaliana* Col-0), and Nicotiana (*N. benthamiana* and *N. tabacum*) were used in this study. The flowers of *O. fragrans* at three flowering stages (bud stage (S1), initial blossoming stage (S2), and full blossoming stage (S3)) were collected for the determination of endogenous hormones, analysis of volatile compounds, and extraction of total RNA. The blank control buffer and MeJA (100 μM; Yuanye, Shanghai, China) solutions containing 0.02% Silwett-L77 (Yuanye, Shanghai, China) were used for the treatment of *O. fragrans* at the S1 stage. MeJA was dissolved in ethanol, mixed with water, and used as the culture solution. Water containing 0.02% Silwett-L77 and ethanol was used as the control buffer. Endogenous hormone contents in flowers at different developmental stages were quantified using liquid chromatography–tandem mass spectrometry. For volatile compound analysis, headspace collection combined with gas chromatography–mass spectrometry (GC–MS) was employed. Total RNA was extracted via the Trizol method, followed by reverse transcription into complementary DNA (cDNA). The resulting cDNA was used for subsequent gene cloning and quantitative Real-Time-PCR (qRT-PCR) analysis.

### Determination of linalool content

The flowers (0.5 g) of *O. fragrans* was collected and placed into a 20 mL capped solid-phase microextraction (SPME) vial. Volatile compounds were collected using a 50/30 μm divinylbenzene/carboxen/polydimethylsiloxane (DVB/CAR/PDMS) fiber for 50 min. Post-extraction, the SPME fiber was inserted into the GC–MS injection port and desorbed at 250°C for 5 min. The analysis employed splitless injection mode with an injector temperature of 250°C. The oven temperature program was: initial 40°C (2 min hold), 3°C/min ramp to 100°C, followed by 5°C/min ramp to 245°C (5 min hold). Mass spectra were acquired in electron ionization (EI) mode at 70 eV, scanning m/z 50–400. Compound identification was performed using the NIST11 database [38].

### Co-expression network and functional enrichment analysis

The online *O. fragrans* genome data analysis website (https://yanglab.hzau.edu.cn/OfIR/home/) was used to construct the gene co-expression network of *OfMYB21* and *OfJAZ3* to determine the molecular regulatory network involved in OfMYB21 and OfJAZ3. All genes (Supplementary Tables S1 and S2) co-expressed with *OfMYB21* and *OfJAZ3* were screened out through the tips of the website, and the KEGG enrichment of these genes were analyzed according to the tools provided by the online website [7].

### Plant transformation

Using the cDNA isolated from the flowers of *O. fragrans* as a template, the coding sequences of *OfMYB21* and *OfJAZ3* were cloned separately. The cloned sequences were then ligated into the overexpression vector containing the CaMV35S promoter. The constructed recombinant plasmids were transformed into *Agrobacterium tumefaciens* GV3101 and used to transform *A. thaliana* via floral dip infiltration. The transformed *A. thaliana* seeds were collected and planted on medium containing hygromycin B (Hyg) to screen for resistant plants [39]. In addition, the overexpression of transgenic *N. tabacum* plants were obtained (Supplementary Fig. S4) using the leaf disc transformation method, and the method was based on published papers [40]. All primers used for the experiment are included in the supplementary document (Supplementary Table S3). Flowers (1 g each) were collected from WT and transgenic *A. thaliana* plants, respectively; similarly, leaves (1 g each) were collected from WT and transgenic *N. tabacum* plants, respectively. The collected flowers or leaves were placed in a mortar, rapidly frozen with liquid nitrogen, and then ground into a fine powder. This powder was transferred to a 20-mL capped solid-phase microextraction (SPME) vial, and volatile compounds were collected using headspace sampling coupled with solid-phase microextraction (SPME) with a 50/30 μm DVB/CAR/PDMS fiber.

### Transient transformation of *O. fragrans* flowers

Transient transformation of *O. fragrans* flowers was performed via vacuum infiltration [41]. *O. fragrans* flowers were placed in a conical flask containing *A. tumefaciens* resuspension and subjected to vacuum infiltration for approximately 10 min. The flowers were then rinsed 2–3 times with 1/2 MS liquid medium to remove residual bacterial solution, transferred to petri dishes, and fully immersed in fresh 1/2 MS liquid medium. After 12 h of dark incubation, the petals were moved to light conditions. Samples were collected 36–48 h post-infiltration to assess target gene expression and volatile compound.

### qRT-PCR assays

Total RNA was extracted from plants using the Trizol method [42]. The extracted RNA was reverse-transcribed into cDNA using the HiScript III All-in-one RT SuperMix Perfect for qPCR kit. Specific primers for qRT-PCR were designed using Primer 5 software, with *OfACT*, *Atactin8* and *NtEF1-α* selected as the reference gene. qRT-PCR analysis was performed using ChamQ Universal SYBR qPCR Master Mix (Vazyme, Nanjing, China). The coding sequences of all genes can be downloaded from the supplementary files (Supplementary Table S4).

### Dual-luciferase assay

The coding sequence of *OfMYB21* or *OfJAZ3* was cloned into the pGreenII 62-SK vector, and the promoter sequence of *OfTPS2* was amplified and ligated into the pGreenII 0800-LUC vector [17]. The constructed effector vector and reporter vector were separately transformed into *A. tumefaciens* GV3101 strain for cultivation. Subsequently, the *A. tumefaciens* cells carrying the effector and reporter vectors were mixed at a 1:1 ratio and resuspended in infiltration buffer to a final OD_600_ of 1.0, followed by 3 h of dark incubation for *N. benthamiana* infiltration. After 3 days of cultivation, the transformed *N. benthamiana* leaves were assayed using a firefly luciferase potassium salt solution, and the activities of luciferase (LUC) and Renilla luciferase (REN) were measured.

### Electrophoretic mobility shift assay

The biotin-labeled probe containing the *OfTPS2* promoter fragment was synthesized [17]. GST-tagged pGEX-6P-1-OfMYB21 and pGEX-6P-1-OfJAZ3 proteins were expressed in *Escherichia coli* strain BL21 and purified using GST Sep Glutathione Magnetic Beads (Yesheng Biotech, Cat. 20562ES03). Electrophoretic mobility shift assay (EMSA) experiments were performed using the chemiluminescent EMSA kit (Beyotime Biotechnology, China) following the manufacturer’s protocol.

### Subcellular localization

The coding sequence of *OfCOI1* or *OfJAZ3* was cloned and inserted into the pCAMBIA1305 vector containing a GFP tag. The constructed expression vector was then transformed into *N. benthamiana* leaves using *A. tumefaciens*-mediated transient transformation. After 40–44 h post-transformation, GFP fluorescence signals were observed using a confocal laser scanning microscope system.

### Split luciferase assays

The coding sequences of *OfMYB21* and *OfCOI1* were cloned and individually inserted into the pCAMBIA1300-nLUC vector, while the *OfJAZ3* coding sequence was cloned into the pCAMBIA1300-cLUC vector. Subsequently, the constructed recombinant plasmids were separately transformed into *A. tumefaciens* strain GV3101 and cultured. The *A. tumefaciens* cultures carrying OfMYB21-nLUC and OfCOI1-nLUC recombinant plasmids were each mixed 1:1 with *A. tumefaciens* harboring OfJAZ3-cLUC, resuspended in infiltration buffer (10 mM MES, 10 mM MgCl_2_, and 150 μM AS) to a final OD_600_ of 1.0, and incubated in the dark for 3 h before *N. benthamiana* leaves infiltration. Luciferase activity was detected using a potassium luciferin solution and a chemiluminescence imaging system (Tanon 5200, Tanon, China).

### Bimolecular fluorescence complementation assay

The coding sequences of *OfCOI1* and *OfJAZ3* were cloned into cYFP and nYFP vectors, respectively. The resulting recombinant plasmids were transformed into *A. tumefaciens* strain GV3101 and cultured. Transient expression in *N. benthamiana* leaves was performed using the method described above, and YFP fluorescence signals were detected by confocal laser scanning microscopy.

### GST pull-down assay

The coding sequence of *OfCOI1* was inserted into the pGEX-6P-1 vector containing a GST tag. Similarly, the *OfJAZ3* coding sequence was cloned into the pET-30a vector with a His-tag. The constructed recombinant plasmids were individually transformed into *E. coli* BL21 cells, and fusion protein expression was induced by adding 0.5 mmol/L isopropyl β-D-1-thiogalactopyranoside (IPTG) to the culture medium. The GST-tagged fusion protein was purified using commercially available GSTSep Glutathione MagBeads (Yisheng Biotechnology, Shanghai, China) according to the manufacturer’s protocol. Pull-down assays were performed following the kit instructions.

### Protein degradation assay

The *N. benthamiana* leaves were infiltrated with *A. tumefaciens* strains harboring 35S::OfJAZ3-GFP. After infiltration 50 h, 3 g of *A. tumefaciens*-infiltrated leaves was collected. The total protein was extracted and incubated at 22°C for indicated time periods without or with 100 μM MeJA, then separated by SDS-PAGE, transferred to PVDF membrane, and detected with GFP antibody. For the detection of GFP fluorescence signals in root tip cells of 35S::OfJAZ3-GFP transgenic *A. thaliana* plants under JA solution treatment, as well as the processing of experimental materials, we followed the detailed procedures described in previously published literature [43].

To investigate the regulatory role of OfCOI1 on OfJAZ3 protein abundance, total proteins were extracted from *N. benthamiana* leaves transiently expressing 35S::OfCOI1-GFP. The recombinant His-OfJAZ3 protein obtained was added to equal amounts of total protein extracts and incubated for 0, 1, 2, 3, 4, and 5 h. Equal volumes of cell lysates were treated with 50 μM MG132. Finally, 2 × SDS loading buffer was added, and samples were boiled at 100°C for 10 min to terminate the reactions. The amount of His-OfJAZ3 protein was then assessed by immunoblotting using His antibody.

### Ubiquitination assay

The ubiquitination assay was conducted with reference to published papers [44]. Using the *Agrobacterium*-mediated transformation method, the constructed 35S::OfJAZ3-GFP recombinant vector was expressed either alone or co-expressed with the 35S::OfCOI1-GFP recombinant vector in *N. benthamiana* leaves. After dark-culturing the transformed tobacco plants for 12 h, they were transferred to light conditions and cultured for 2 days. Leaf samples were collected following a 12-h treatment with 50 μM MG132. For immunoprecipitation, GFP-tagged protein magnetic beads were incubated with total protein extracted from *N. benthamiana* leaves for 2–3 h. Non-specific proteins bound to the magnetic beads were washed away using washing buffer, and the proteins were then eluted with 1 × SDS loading buffer. Subsequently, immunoblot analysis was performed using anti-ubiquitin antibodies and anti-GFP antibodies, respectively.

## Supplementary Material

Web_Material_uhaf321

## Data Availability

The data supporting the findings of this study are available within the paper figures and the Supplementary Information.
