# Supplementary material for: The OfJAZ3-OfMYB21 complex mediates jasmonic acid signaling pathway to regulate linalool biosynthesis in Osmanthus fragrans
Source: Hortic Res. 2025 Nov 25;13(3):uhaf321. doi: 10.1093/hr/uhaf321 (PMC12963045; doi:10.1093/hr/uhaf321)
Supplement: Web_Material_uhaf321 [file web_material_uhaf321.zip › 2025.10.22-Supplementary figure.docx]

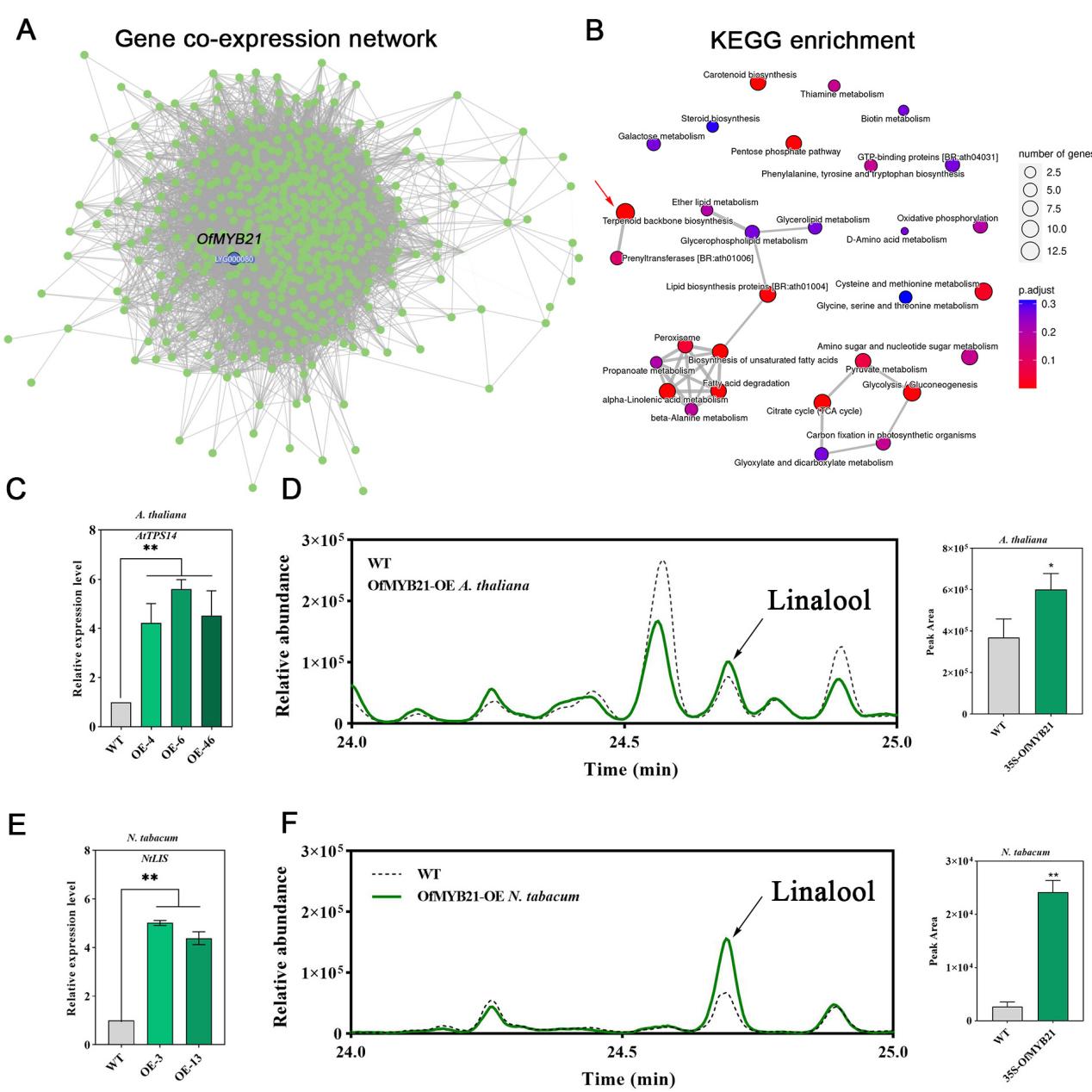


**Fig. S1** OfMYB21 acts as a positive regulator of linalool biosynthesis. (A) Gene co-expression network involving *OfMYB21*. (B) KEGG pathway enrichment analysis for genes co-expressed with *OfMYB21*. (C) Detection of the expression levels of the linalool synthase gene *AtTPS14* in WT and *OfMYB21*-overexpressing transgenic *A. thaliana* plants. (D) Comparison of linalool abundance between WT and *OfMYB21*-overexpressing transgenic *A. thaliana* plants. (E) Detection of the expression levels of the linalool synthase gene *NtLIS* in WT and *OfMYB21*-overexpressing transgenic *N. tabacum* plants. (F) Comparison of linalool abundance between WT and *OfMYB21*-overexpressing transgenic *N. tabacum* plants.


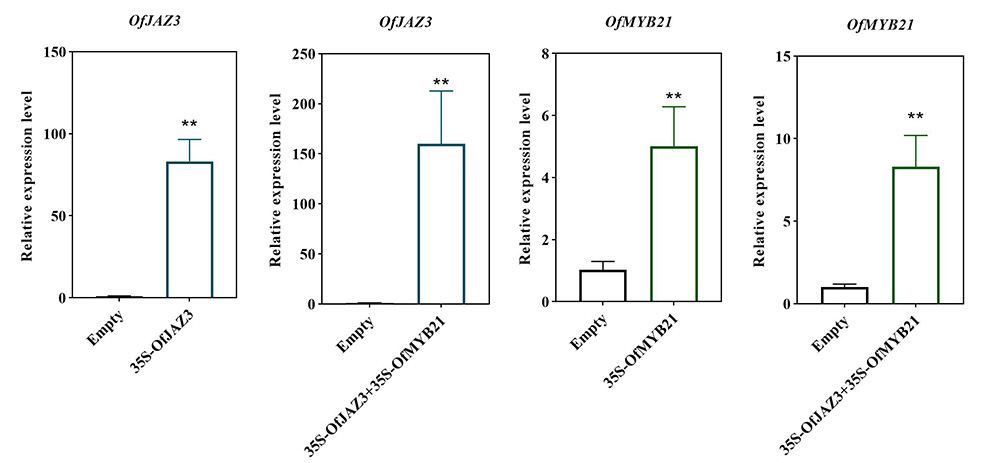


**Fig. S2** qRT-PCR was used to verify the transient expression levels of *OfMYB21* and *OfJAZ3* when expressed individually or co-expressed in *O. fragrans* flowers.


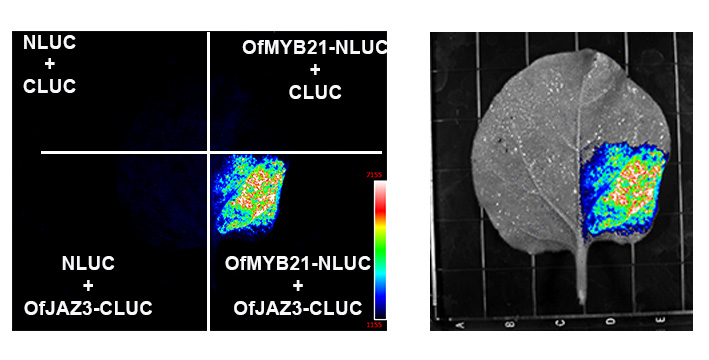


**Fig. S3** The Split-LUC assay was used to verify the interaction between OfMYB21 and OfJAZ3.


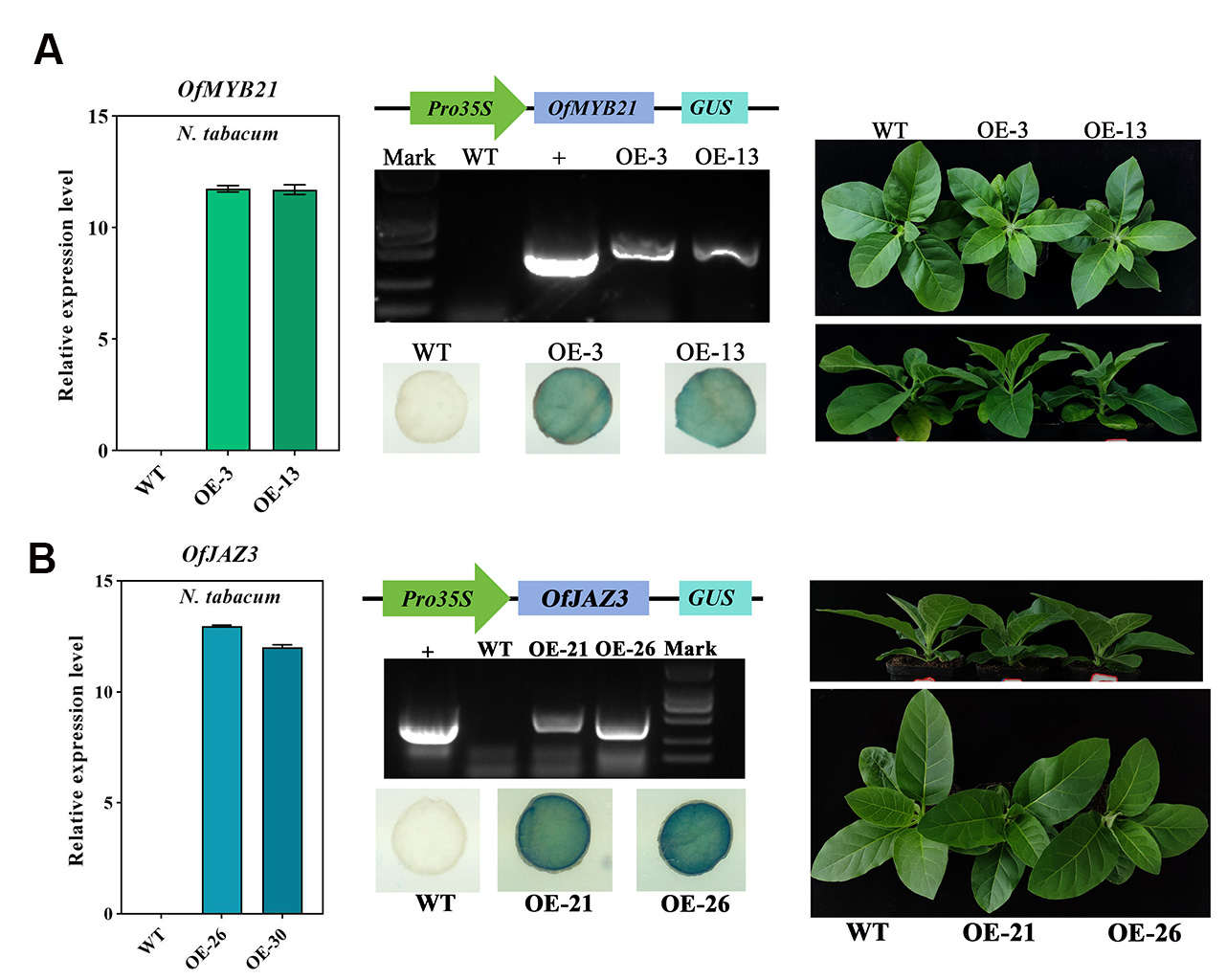


**Fig. S4** Validation of two *OfMYB21/OfJAZ3*-overexpressing transgenic *N. tabacum* lines.
